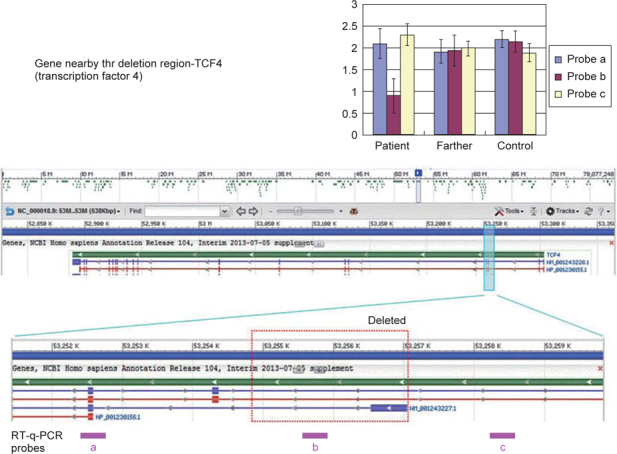# A case of Pitt-Hopkins syndrome presented with Angelman-like syndromic phenotypes

**DOI:** 10.7603/s40681-016-0025-1

**Published:** 2016-11-19

**Authors:** Syuan-Yu Hong, I-Ching Chou, Wei-De Lin, Fuu-Jen Tsai

**Affiliations:** 1 Department of Pediatrics, Children’s Hospital, China Medical University Hospital, Taichung, 404, Taiwan; 2 Department of Medical Research, China Medical University and Hospital, Taichung, 404, Taiwan; 3 School of Post-baccalaureate Chinese Medicine, China Medical University, Taichung, 404, Taiwan; 4 Department of Pediatrics, China Medical University Hospital, No. 2 Yuh-Der Road, Taichung, 404, Taiwan

**Keywords:** Pitt-Hopkins syndrome, Angelman-like syndrome, TCF4 gene

## Abstract

Pitt-Hopkins syndrome (PTHS), caused by a TCF4 gene mutation, is a condition characterized by intellectual disability and developmental delay, breathing anomalies, epilepsy, and distinctive facial dysmorphism [1]. Its diverse clinical appearance causes pediatricians to confuse it with Angelman syndrome, which is considered one of the family members of Angelman-like syndrome. Herein, we report on a 4 y/o boy with PTHS and discuss its similarities and differences with Angelman syndrome. In doing so we hope to provide a feasible pathway to diagnose rare diseases, especially Angelman-like syndrome.

## 1. Introduction

Pitt-Hopkins syndrome (PTHS), a condition characterized by intellectual disability and developmental delay, breathing anomalies, possible occurrence of epilepsy, and distinctive facial dysmorphism, is caused by a *TCF4* (transcription cell factor 4) gene mutation [1-5]. However, PTHS could also be considered a part of the Angelman-like syndrome category [6-8]. Herein, we present a case of Pitt-Hopkins syndrome with Angelman-like syndrome phenotypes.

## 2. Case report

The 4y/o boy was born from healthy, unrelated Taiwanese parents. His mother had regular prenatal examinations and was uneventful throughout her pregnancy. After birth, his head circumference was 29 cm (< 2 standard deviations), his birth weight was 2250g (<10^th^ percentile), and his birth height was 41cm (<10^th^ percentile). During the first year of his life, a severe developmental delay became increasingly apparent. He could not sit until 12 months, was unable to walk at that period of time, and his language abilities had not developed. He was a cheerful boy with some stereotypical hand movements. At 2 years and 8 months, unexplained tachypnea was noted, which occasionally brought out a breathing arrest. At the same time, brief seizures characterized by cyanosis, eye staring, and loss of muscle tone with or without apnea were frequently occurring. A complete metabolic screening and chromosome analysis revealed nothing abnormal. Neuroimaging (including brain MRI and CT), ocular examination, and sonography for his abdomen and heart all presented negative results.

**Figure 1:**
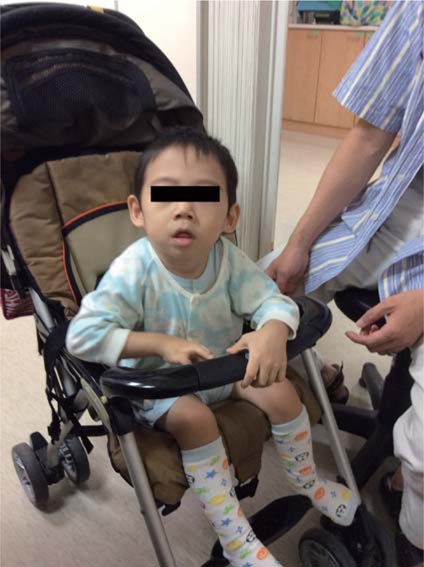

When he was 3 years and 8 months old he visited our outpatient clinic, and at that time his distinctive facial features were impressive: microcephaly (head circumference was 45 cm, <5^th^ percentile), a weight of 11.5kg (<10^th^ percentile), a short stature (height 92 cm, <10^th^ percentile), strabismus, relatively small hands and feet, a pronounced upper lip with a Cupid’s bow shape, a mouth with full lips that he was unable to close, and thick and cup-shaped ears.(Fig. 1). The awake-and-sleep EEG revealed epileptiform activities. He then received anti-epileptic therapy with levetiracetam and obtained a significant control over his seizures. A microarray analysis was conducted and a single-copy loss of 2 kb (chr18:53,254,861–53,257,075) was found. To validate the microarray data, real-time quantitative PCR (RT-q-PCR) was used and TCF4 gene deletion was identified. (Fig. 2)

## 3. Discussion

Pitt-Hopkins syndrome is a very rare condition, with less than 600 cases being reported in the entire world so far. To our knowledge, this is the first Taiwanese case of PTHS diagnosed at the molecular level. However, our aim is not to stress how rare it is. Instead, we hope to provide a feasible pathway to diagnose rare diseases such as PTHS, especially those with Angelman-like syndromes.

The first time we saw this patient, he had severe developmental delays, absence of speech; involuntary movements, a distinct face with a wide mouth, and unprovoked episodes of laughter and smiling, and all of this led us to a diagnosis of Angelman syndrome/ Angelman-like syndrome. Before any molecular analysis is carried out, it is crucial to understand which disorders have symptoms similar to those of Angelman syndrome [6-8]. Besides Angelman syndrome (AS), which is caused by a deficiency of the UBE3A gene in the brain [6, 9-12], these Angelman-like syndromes can be derived from chromosomal microdeletion/ microduplication syndromes, or single-gene disorders. The former include Phelan–McDermid syndrome (chromosome 22q13.3 deletion), MBD5 haploinsufficiency syndrome (chromosome 2q23.1 deletion), and KANSL1 haploinsufficiency syndrome (chromosome 17q21.31 deletion). The latter, the single-gene disorders, include Pitt–Hopkins syndrome (TCF4), Christianson syndrome (SLC9A6), Mowat–Wilson syndrome (ZEB2), Kleefstra syndrome (EHMT1), and Rett syndrome (MECP2). They also include disorders due to mutations in HERC2, adenylosuccinaselyase (ADSL), CDKL5, FOXG1, MECP2 (duplications), MEF2C, and ATRX [12-17]. The aforementioned diseases could evidence the same clinical features in variation and overlapping, which makes finding the correct diagnosis more challenging and confusing. For example, almost all patients with Angelman-like syndromes could have seizures and speech impairments or impediemnts; patients with AS, PTHS, Christianson syndrome, Rett syndrome, and Mowat–Wilson syndrome could all have happy dispositions; Rett and PTHS both could have breathing problems like hyperventilation or apnea [18, 19], and the list goes on. More elusively, even the symptoms specifically belonging to a particular disease are not guaranteed to appear in a patient with said particular disease, and even if or when they do appear, they may not do so until later in life [19, 20].

From this case, a single-copy loss of 2 kb (chr18:53,254,861– 53,257,075) was characterized via microarray analysis. To confirm the deletion, quantitative real-time PCR was performed and TCF4 haploinsufficiency was eventually confirmed. Hence, we believe that PTHS should be taken into account when diagnosing patients with facial dysmorphism, profound intellectual disability, epilepsy, and breathing anomalies. Furthermore, a gene sequencing and analysis panel for “Angelman-like syndromes” that contain those associated genes should be pursued for a more accurate diagnosis.

## 4. Conclusion

PTHS should be suspected on the strength of clinical findings like severe development delay, breathing anomalies, absent speech ability, and special facial appearance. Moreover, we believe that PTHS should also be taken into account in patients presenting an Angelman-like syndrome. To increase the accuracy of diagnosis, it is necessary to build a methodical molecular analysis for Angelman- like syndromes. And hopefully, such molecular analysis will become a conventional examination in future.

**Figure 2:**